# 1,3-Diammonio-1,2,3-tride­oxy-*cis*-inositol sulfate

**DOI:** 10.1107/S1600536812016029

**Published:** 2012-04-18

**Authors:** Christian Neis, Günter J. Merten, Kaspar Hegetschweiler

**Affiliations:** a Fachrichtung Chemie, Universität des Saarlandes, Postfach 151150, D-66041 Saarbrücken, Germany

## Abstract

In the crystal structure of the title compound, C_6_H_16_N_2_O_3_^2+^·SO_4_^2−^, each cation forms three O—H⋯O and five N—H⋯O hydrogen bonds to six neighbouring sulfate anions. In addition, interlinking of the cations by N—H⋯O interactions is also observed. The cyclo­hexane ring adopts a chair conformation with two axial hy­droxy groups. Although the separation of 2.928 Å is almost ideal for a hydrogen bond, intra­molecular hydrogen bonding between these two hy­droxy groups is not observed.

## Related literature

The synthesis of the chloride salt, as well as formation of a Cu^II^ complex of 1,3-diamino-1,2,3-tride­oxy-*cis*-inositol, was reported by Merten *et al.* (2012 ▶). A crystal structure deter­min­ation of the chloride salt was performed by Neis *et al.* (2012 ▶). The importance of intra­molecular hydrogen bonding in *syn*-1,3,5-tris­ubstituted cyclo­hexane derivatives has been discussed by Gencheva *et al.* (2000 ▶), Kramer *et al.* (1998 ▶), Kuppert *et al.* (2006 ▶), and Neis *et al.* (2010 ▶). Puckering parameters were calculated according to Cremer & Pople (1975 ▶). For the treatment of hydrogen atoms in *SHELXL*, see: Müller *et al.* (2006 ▶).
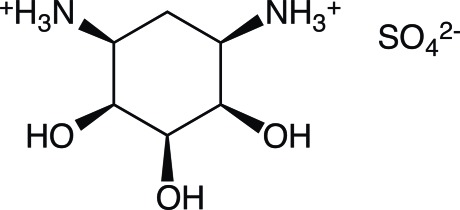


## Experimental

### 

#### Crystal data

C_6_H_16_N_2_O_3_^2+^·SO_4_^2−^
*M* *_r_* = 260.27
Monoclinic, 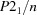

*a* = 9.2151 (18) Å
*b* = 6.6673 (13) Å
*c* = 17.267 (4) Å
β = 101.46 (3)°
*V* = 1039.7 (4) Å^3^
*Z* = 4
Mo *K*α radiation
μ = 0.34 mm^−1^
*T* = 200 K
0.30 × 0.20 × 0.15 mm

#### Data collection

Stoe IPDS image plate diffractometer
6961 measured reflections
1779 independent reflections
1685 reflections with *I* > 2σ(*I*)
*R*_int_ = 0.035

#### Refinement

*R*[*F* ^2^ > 2σ(*F* ^2^)] = 0.026
*wR*(*F* ^2^) = 0.066
*S* = 1.06
1779 reflections
172 parameters
9 restraints
H atoms treated by a mixture of independent and constrained refinement
Δρ_max_ = 0.27 e Å^−3^
Δρ_min_ = −0.29 e Å^−3^

### 

Data collection: *IPDS Software* (Stoe & Cie, 1997 ▶); cell refinement: *IPDS Software*; data reduction: *IPDS Software*; program(s) used to solve structure: *SHELXS97* (Sheldrick, 2008 ▶); program(s) used to refine structure: *SHELXL97* (Sheldrick, 2008 ▶); molecular graphics: *DIAMOND* (Brandenburg, 2011 ▶); software used to prepare material for publication: *SHELXL97* and *PLATON* (Spek, 2009 ▶).

## Supplementary Material

Crystal structure: contains datablock(s) global, I. DOI: 10.1107/S1600536812016029/bx2402sup1.cif

Structure factors: contains datablock(s) I. DOI: 10.1107/S1600536812016029/bx2402Isup2.hkl

Additional supplementary materials:  crystallographic information; 3D view; checkCIF report

## Figures and Tables

**Table 1:** Hydrogen-bond geometry (Å, °)

*D*—H⋯*A*	*D*—H	H⋯*A*	*D*⋯*A*	*D*—H⋯*A*
N1—H1*A*⋯O4^i^	0.87 (1)	2.09 (2)	2.9292 (17)	163 (2)
N1—H1*B*⋯O3^ii^	0.87 (2)	2.11 (2)	2.8125 (18)	138 (2)
N1—H1*C*⋯O7^iii^	0.85 (2)	2.04 (2)	2.882 (2)	170 (2)
N5—H5*A*⋯O5	0.89 (2)	1.97 (2)	2.8538 (17)	173 (2)
N5—H5*B*⋯O8^iv^	0.86 (1)	2.02 (2)	2.8530 (18)	162 (2)
N5—H5*C*⋯O7^ii^	0.87 (2)	2.29 (2)	3.0852 (18)	153 (2)
N5—H5*C*⋯O6^ii^	0.87 (2)	2.34 (2)	3.0856 (19)	144 (2)
O4—H4*O*⋯O6	0.85 (2)	1.86 (2)	2.7017 (16)	177 (2)
O2—H2*O*⋯O8^v^	0.86 (2)	1.97 (2)	2.8161 (15)	170 (2)
O3—H3*O*⋯O7^vi^	0.86 (2)	1.87 (2)	2.7149 (16)	167 (2)

Symmetry codes: (i) 
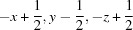
; (ii) 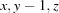; (iii) 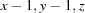; (iv) 
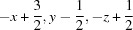
; (v) 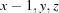; (vi) 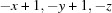.

**Fig. 1.**
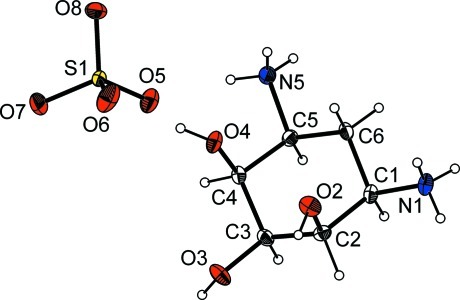
Molecular structure of the title compound with numbering scheme and displacement ellipsoids drawn at the 50% probability level.

**Fig. 2.**
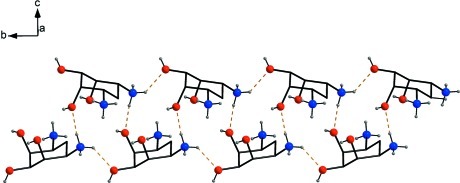
The double chain structure which is formed by hydrogen bonding between dication entities (ball and stick model).

## supplementary crystallographic information

### Comment

Due to the versatile metal- and hydrogen-binding properties, 1,3-diamino-1,2,3-trideoxy-*cis*-inositol has been found to be a promising building block for the construction of polynuclear metal complexes and extended hydrogen bonded networks (Merten *et al.*, 2012). The crystal structure of a corresponding hydrochloride C_6_H_14_N_2_O_3_^.^2HCl has recently been reported (Neis *et al.*, 2012). Similar to this chloride salt, the title compound contains 1,3-diammonio-1,2,3-trideoxy-*cis*-inositol dications with the cyclohexane ring adopting an almost ideal chair conformation (puckering parameters: Q = 0.59 Å, θ = 178.3 °, φ = 89.0 °). In both salts, the cation has the same form with the two ammonium groups and one of the hydroxy groups in equatorial and the remaining two hydroxy groups in axial position. In the title compound, each cation is hydrogen-bonded to six sulfate counter ions by O—H···O and N—H···O interactions, one of the latter is bifurcated. These cation···anion interactions constitute an extended three-dimensional network. Direct cation···cation hydrogen bonding is also observed: One of the ammonium groups of each cation donates a hydrogen atom to the equatorial hydroxy group of a neighbouring cation and another hydrogen atom to the axial hydroxy group of an additional neighbour. Each cation is thus connected to a total of four neighbouring cations and these interactions generate double chains, which are oriented parallel to the crystallographic *b* axis. As already observed for the chloride salt, all O—H and N—H groups act as hydrogen donors, however, one of the axial hydroxy groups does not accept any hydrogen atom. We explain these observations by the well established stronger steric encumbrance of axial substituents. It is again worthy to note that the non-accepting axial hydroxy group does not form an intramolecular O—H···O hydrogen bond, even though the O···O separation of 2.928 Å between the two axial oxygen atoms corresponds almost ideally to the value required for such an interaction. For corresponding structures with three axial hydroxy or amino groups in a *syn*-1,3,5-triaxial arrangement, it appears, however, that the formation of such intramolecular hydrogen bonds is often a prerequisite for a stable cyclohexane chair (Gencheva *et al.*, 2000; Kramer *et al.*, 1998; Kuppert *et al.*, 2006). The non-observance of such a hydrogen bond in the title compound further supports the conclusion that this type of interactions would be of minor importance in molecules having only two hydroxy groups in a 1,3-*syn*-axial arrangement.

### Experimental

The title compound has been obtained following the protocol given by Merten *et al.* (2012). ^1^H-NMR and ^13^C-NMR properties are identical with the chloride salt (Neis *et al.*, 2012). Single crystals were grown from an aqueous solution (pH 2) by slow evaporation at 298 K.

### Refinement

All non-hydrogen atoms were refined using anisotropic displacement parameters. Hydrogen atoms were treated as recommended by Müller *et al.* (2006): A riding model was used for C-bonded hydrogen atoms. The positional parameters of the O- and N-bonded hydrogen atoms were refined using isotropic displacement parameters which were set to 1.5×*U*_eq_ of the pivot atom. In addition, restraints of 0.84 and 0.88 Å were used for the O—H and N—H distances, respectively.

### Crystal data

**Table:**

C_6_H_16_N_2_O_3_^2+^·SO_4_^2^^−^	*F*(000) = 552
*M**_r_* = 260.27	*D*_x_ = 1.663 Mg m^−^^3^
Monoclinic, *P*2_1_/*n*	Mo *K*α radiation, λ = 0.71073 Å
Hall symbol: -P 2yn	Cell parameters from 3837 reflections
*a* = 9.2151 (18) Å	θ = 2.6–25.0°
*b* = 6.6673 (13) Å	µ = 0.34 mm^−^^1^
*c* = 17.267 (4) Å	*T* = 200 K
β = 101.46 (3)°	Prism, colourless
*V* = 1039.7 (4) Å^3^	0.30 × 0.20 × 0.15 mm
*Z* = 4	

### Data collection

**Table:**

Stoe IPDS image plate diffractometer	1685 reflections with *I* > 2σ(*I*)
Radiation source: fine-focus sealed tube	*R*_int_ = 0.035
Graphite monochromator	θ_max_ = 25.0°, θ_min_ = 2.3°
phi scans	*h* = −10→10
6961 measured reflections	*k* = −7→7
1779 independent reflections	*l* = −20→20

### Refinement

**Table:**

Refinement on *F*^2^	Primary atom site location: structure-invariant direct methods
Least-squares matrix: full	Secondary atom site location: difference Fourier map
*R*[*F*^2^ > 2σ(*F*^2^)] = 0.026	Hydrogen site location: inferred from neighbouring sites
*wR*(*F*^2^) = 0.066	H atoms treated by a mixture of independent and constrained refinement
*S* = 1.06	*w* = 1/[σ^2^(*F*_o_^2^) + (0.0259*P*)^2^ + 0.7072*P*] where *P* = (*F*_o_^2^ + 2*F*_c_^2^)/3
1779 reflections	(Δ/σ)_max_ = 0.001
172 parameters	Δρ_max_ = 0.27 e Å^−^^3^
9 restraints	Δρ_min_ = −0.29 e Å^−^^3^

### Special details

**Table:**

Geometry. All e.s.d.'s (except the e.s.d. in the dihedral angle between two l.s. planes) are estimated using the full covariance matrix. The cell e.s.d.'s are taken into account individually in the estimation of e.s.d.'s in distances, angles and torsion angles; correlations between e.s.d.'s in cell parameters are only used when they are defined by crystal symmetry. An approximate (isotropic) treatment of cell e.s.d.'s is used for estimating e.s.d.'s involving l.s. planes.
Refinement. Refinement of *F*^2^ against ALL reflections. The weighted *R*-factor *wR* and goodness of fit *S* are based on *F*^2^, conventional *R*-factors *R* are based on *F*, with *F* set to zero for negative *F*^2^. The threshold expression of *F*^2^ > σ(*F*^2^) is used only for calculating *R*-factors(gt) *etc*. and is not relevant to the choice of reflections for refinement. *R*-factors based on *F*^2^ are statistically about twice as large as those based on *F*, and *R*- factors based on ALL data will be even larger.

### Fractional atomic coordinates and isotropic or equivalent isotropic displacement parameters (Å^2^)

**Table:**

	*x*	*y*	*z*	*U*_iso_*/*U*_eq_	
C5	0.44604 (15)	−0.0876 (2)	0.10574 (8)	0.0129 (3)	
H5	0.4373	−0.1399	0.0507	0.015*	
C6	0.35712 (15)	−0.2238 (2)	0.15088 (8)	0.0149 (3)	
H6A	0.3961	−0.3624	0.1521	0.018*	
H6B	0.3684	−0.1762	0.2061	0.018*	
C2	0.12716 (15)	−0.0111 (2)	0.10691 (8)	0.0139 (3)	
H2	0.0241	−0.0171	0.0749	0.017*	
C1	0.19415 (15)	−0.2228 (2)	0.11143 (8)	0.0136 (3)	
H1	0.1839	−0.2768	0.0566	0.016*	
C3	0.22081 (16)	0.1251 (2)	0.06417 (8)	0.0137 (3)	
H3	0.2117	0.0722	0.0092	0.016*	
C4	0.38668 (15)	0.1297 (2)	0.10194 (8)	0.0126 (3)	
H4	0.4396	0.2105	0.0674	0.015*	
N1	0.11358 (15)	−0.3591 (2)	0.15750 (8)	0.0171 (3)	
H1A	0.124 (2)	−0.325 (3)	0.2070 (9)	0.026*	
H1B	0.142 (2)	−0.481 (2)	0.1514 (10)	0.026*	
H1C	0.0204 (17)	−0.360 (3)	0.1393 (10)	0.026*	
N5	0.60555 (14)	−0.09248 (19)	0.14663 (8)	0.0151 (3)	
H5A	0.6514 (19)	0.006 (3)	0.1269 (10)	0.023*	
H5B	0.612 (2)	−0.084 (3)	0.1971 (9)	0.023*	
H5C	0.642 (2)	−0.208 (2)	0.1385 (10)	0.023*	
O4	0.41236 (12)	0.21890 (16)	0.17886 (6)	0.0177 (2)	
H4O	0.4859 (19)	0.294 (3)	0.1786 (11)	0.027*	
O2	0.12055 (12)	0.05338 (16)	0.18490 (6)	0.0188 (2)	
H2O	0.0491 (19)	0.137 (3)	0.1815 (11)	0.028*	
O3	0.15853 (12)	0.32218 (16)	0.05817 (6)	0.0206 (2)	
H3O	0.171 (2)	0.369 (3)	0.0137 (9)	0.031*	
S1	0.77047 (4)	0.40360 (5)	0.132375 (19)	0.01264 (13)	
O7	0.80382 (13)	0.58885 (16)	0.09000 (6)	0.0268 (3)	
O8	0.90424 (11)	0.34825 (16)	0.19086 (6)	0.0201 (2)	
O5	0.72807 (12)	0.23732 (16)	0.07600 (6)	0.0221 (3)	
O6	0.65015 (12)	0.45266 (18)	0.17466 (7)	0.0294 (3)	

### Atomic displacement parameters (Å^2^)

**Table:**

	*U*^11^	*U*^22^	*U*^33^	*U*^12^	*U*^13^	*U*^23^
C5	0.0114 (7)	0.0124 (7)	0.0148 (7)	−0.0003 (5)	0.0022 (5)	−0.0010 (5)
C6	0.0148 (7)	0.0096 (7)	0.0200 (7)	−0.0004 (5)	0.0027 (5)	0.0025 (5)
C2	0.0112 (7)	0.0156 (7)	0.0142 (6)	0.0004 (5)	0.0008 (5)	−0.0008 (5)
C1	0.0151 (7)	0.0118 (7)	0.0146 (6)	−0.0038 (5)	0.0043 (5)	−0.0007 (5)
C3	0.0165 (7)	0.0106 (7)	0.0137 (6)	0.0026 (5)	0.0024 (5)	0.0004 (5)
C4	0.0150 (7)	0.0104 (7)	0.0131 (6)	−0.0017 (5)	0.0044 (5)	−0.0001 (5)
N1	0.0178 (7)	0.0142 (6)	0.0205 (7)	−0.0052 (5)	0.0067 (5)	−0.0017 (5)
N5	0.0129 (6)	0.0132 (6)	0.0191 (6)	0.0008 (5)	0.0032 (5)	−0.0012 (5)
O4	0.0199 (5)	0.0158 (5)	0.0179 (5)	−0.0065 (4)	0.0045 (4)	−0.0047 (4)
O2	0.0209 (6)	0.0195 (5)	0.0172 (5)	0.0050 (4)	0.0069 (4)	−0.0003 (4)
O3	0.0269 (6)	0.0136 (5)	0.0223 (5)	0.0083 (4)	0.0073 (4)	0.0046 (4)
S1	0.0110 (2)	0.01085 (19)	0.0156 (2)	−0.00160 (12)	0.00125 (13)	0.00023 (12)
O7	0.0345 (7)	0.0183 (6)	0.0244 (6)	−0.0064 (5)	−0.0017 (5)	0.0082 (4)
O8	0.0183 (5)	0.0207 (5)	0.0193 (5)	0.0035 (4)	−0.0009 (4)	0.0023 (4)
O5	0.0255 (6)	0.0209 (6)	0.0205 (5)	−0.0077 (4)	0.0055 (4)	−0.0057 (4)
O6	0.0168 (6)	0.0289 (6)	0.0455 (7)	−0.0041 (5)	0.0134 (5)	−0.0149 (5)

### Geometric parameters (Å, º)

**Table:**

C5—N5	1.4993 (18)	C4—O4	1.4312 (17)
C5—C6	1.5355 (19)	C4—H4	1.0000
C5—C4	1.5454 (19)	N1—H1A	0.870 (14)
C5—H5	1.0000	N1—H1B	0.867 (15)
C6—C1	1.521 (2)	N1—H1C	0.853 (15)
C6—H6A	0.9900	N5—H5A	0.885 (15)
C6—H6B	0.9900	N5—H5B	0.864 (14)
C2—O2	1.4265 (17)	N5—H5C	0.866 (15)
C2—C1	1.536 (2)	O4—H4O	0.845 (15)
C2—C3	1.5386 (19)	O2—H2O	0.855 (15)
C2—H2	1.0000	O3—H3O	0.856 (15)
C1—N1	1.4985 (18)	S1—O5	1.4758 (11)
C1—H1	1.0000	S1—O8	1.4768 (11)
C3—O3	1.4297 (17)	S1—O6	1.4802 (12)
C3—C4	1.538 (2)	S1—O7	1.4982 (11)
C3—H3	1.0000		
			
N5—C5—C6	108.67 (11)	C2—C3—H3	107.4
N5—C5—C4	110.25 (11)	O4—C4—C3	111.66 (11)
C6—C5—C4	110.85 (11)	O4—C4—C5	110.99 (11)
N5—C5—H5	109.0	C3—C4—C5	108.20 (11)
C6—C5—H5	109.0	O4—C4—H4	108.6
C4—C5—H5	109.0	C3—C4—H4	108.6
C1—C6—C5	110.43 (11)	C5—C4—H4	108.6
C1—C6—H6A	109.6	C1—N1—H1A	113.0 (13)
C5—C6—H6A	109.6	C1—N1—H1B	108.2 (12)
C1—C6—H6B	109.6	H1A—N1—H1B	112.8 (17)
C5—C6—H6B	109.6	C1—N1—H1C	112.2 (13)
H6A—C6—H6B	108.1	H1A—N1—H1C	105.8 (18)
O2—C2—C1	108.74 (11)	H1B—N1—H1C	104.5 (18)
O2—C2—C3	114.09 (11)	C5—N5—H5A	107.5 (12)
C1—C2—C3	107.97 (11)	C5—N5—H5B	109.5 (12)
O2—C2—H2	108.6	H5A—N5—H5B	113.7 (16)
C1—C2—H2	108.6	C5—N5—H5C	108.8 (12)
C3—C2—H2	108.6	H5A—N5—H5C	111.5 (17)
N1—C1—C6	107.99 (11)	H5B—N5—H5C	105.7 (17)
N1—C1—C2	110.35 (12)	C4—O4—H4O	103.2 (12)
C6—C1—C2	112.20 (11)	C2—O2—H2O	107.9 (12)
N1—C1—H1	108.7	C3—O3—H3O	106.1 (13)
C6—C1—H1	108.7	O5—S1—O8	109.81 (7)
C2—C1—H1	108.7	O5—S1—O6	111.42 (6)
O3—C3—C4	111.27 (11)	O8—S1—O6	108.86 (7)
O3—C3—C2	108.78 (11)	O5—S1—O7	110.50 (6)
C4—C3—C2	114.41 (11)	O8—S1—O7	108.39 (6)
O3—C3—H3	107.4	O6—S1—O7	107.77 (7)
C4—C3—H3	107.4		
			
N5—C5—C6—C1	179.85 (11)	O2—C2—C3—C4	65.11 (15)
C4—C5—C6—C1	58.54 (15)	C1—C2—C3—C4	−55.89 (14)
C5—C6—C1—N1	179.52 (11)	O3—C3—C4—O4	58.04 (15)
C5—C6—C1—C2	−58.64 (15)	C2—C3—C4—O4	−65.75 (15)
O2—C2—C1—N1	51.84 (14)	O3—C3—C4—C5	−179.54 (11)
C3—C2—C1—N1	176.11 (10)	C2—C3—C4—C5	56.67 (15)
O2—C2—C1—C6	−68.64 (14)	N5—C5—C4—O4	−53.77 (15)
C3—C2—C1—C6	55.63 (14)	C6—C5—C4—O4	66.61 (14)
O2—C2—C3—O3	−60.00 (15)	N5—C5—C4—C3	−176.61 (11)
C1—C2—C3—O3	179.00 (10)	C6—C5—C4—C3	−56.23 (15)

### Hydrogen-bond geometry (Å, º)

**Table:**

*D*—H···*A*	*D*—H	H···*A*	*D*···*A*	*D*—H···*A*
N1—H1*A*···O4^i^	0.87 (1)	2.09 (2)	2.9292 (17)	163 (2)
N1—H1*B*···O3^ii^	0.87 (2)	2.11 (2)	2.8125 (18)	138 (2)
N1—H1*C*···O7^iii^	0.85 (2)	2.04 (2)	2.882 (2)	170 (2)
N5—H5*A*···O5	0.89 (2)	1.97 (2)	2.8538 (17)	173 (2)
N5—H5*B*···O8^iv^	0.86 (1)	2.02 (2)	2.8530 (18)	162 (2)
N5—H5*C*···O7^ii^	0.87 (2)	2.29 (2)	3.0852 (18)	153 (2)
N5—H5*C*···O6^ii^	0.87 (2)	2.34 (2)	3.0856 (19)	144 (2)
O4—H4*O*···O6	0.85 (2)	1.86 (2)	2.7017 (16)	177 (2)
O2—H2*O*···O8^v^	0.86 (2)	1.97 (2)	2.8161 (15)	170 (2)
O3—H3*O*···O7^vi^	0.86 (2)	1.87 (2)	2.7149 (16)	167 (2)

Symmetry codes: (i) −*x*+1/2, *y*−1/2, −*z*+1/2; (ii) *x*, *y*−1, *z*; (iii) *x*−1, *y*−1, *z*; (iv) −*x*+3/2, *y*−1/2, −*z*+1/2; (v) *x*−1, *y*, *z*; (vi) −*x*+1, −*y*+1, −*z*.
